# Detoxified O-Specific Polysaccharide (O-SP)–Protein Conjugates: Emerging Approach in the *Shigella* Vaccine Development Scene

**DOI:** 10.3390/vaccines10050675

**Published:** 2022-04-24

**Authors:** Dani Cohen, Shiri Meron-Sudai, Anya Bialik, Valeria Asato, Shai Ashkenazi

**Affiliations:** 1 School of Public Health, Sackler Faculty of Medicine, Tel Aviv University, Tel Aviv 69978, Israel; shiri.meron.sudai@gmail.com (S.M.-S.); bialika@tauex.tau.ac.il (A.B.); valeria.asato@gmail.com (V.A.); 2 Adelson School of Medicine, Ariel University, Ariel 40700, Israel; shaias@ariel.ac.il; 3 Schneider Children’s Medical Center, Petach Tikva 49202, Israel

**Keywords:** shigella, conjugate vaccines, ELISA, IgG, correlates of protection

## Abstract

*Shigella* is the second most common cause of moderate to severe diarrhea among children worldwide and of diarrheal disease-associated mortality in young children in low-and middle-income countries. In spite of many years of attempts to develop *Shigella* vaccines, no licensed vaccines are yet available. Injectable conjugate vaccines made of the detoxified lipopolysaccharide (LPS) of *S. flexneri* 2a, *S. sonnei*, and *S. dysenteriae* type 1 covalently bound to protein carriers were developed in the early 1990s by John B. Robbins and Rachel Schneerson at the US National Institutes of Health. This approach was novel for a disease of the gut mucosa, at a time when live, rationally attenuated oral vaccine strains that intended to mimic *Shigella* infection and induce a protective local immune response were extensively investigated. Of keystone support to *Shigella* glycoconjugates development were the findings of a strong association between pre-existent serum IgG antibodies to *S. sonnei* or *S. flexneri* 2a LPS and a lower risk of infection with the homologous *Shigella* serotypes among Israeli soldiers serving in field units. In view of these findings and of the successful development of the pioneering *Haemophilus influenzae* type b conjugate vaccines, it was hypothesized that protective immunity may be conferred by serum IgG antibodies to the O-Specific Polysaccharide (O-SP) following parenteral delivery of the conjugates. *S. sonnei* and *S. flexneri* 2a glycoconjugates induced high levels of serum IgG against the homologous LPS in phase I and II studies in healthy volunteers. The protective efficacy of a *S. sonnei* detoxified LPS-conjugate was further demonstrated in field trials in young adults (74%) and in children older than three years of age (71%), but not in younger ones. The evaluation of the *Shigella* conjugates confirmed that IgG antibodies to *Shigella* LPS are correlates of protection and provided solid basis for the development of a new generation of glycoconjugates and other injectable LPS-based vaccines that are currently in advanced stages of clinical evaluation.

## 1. Introduction

*Shigella* is a leading cause of moderate to severe diarrhea and continues to be associated with a considerable burden of disease across the globe. Shigellosis is highly endemic in low- and middle-income countries (LMICs) where approximately 250 million new cases occur yearly with over 212,000 fatal cases of whom ~63,000 among children less than five years old [1]. Children with shigellosis have an increased risk of persistent diarrhea, malnutrition, and linear growth failure [1,2,3]. *Shigella* is also responsible for around 2 million cases of shigellosis in high-income countries [1,4] where at-risk populations include children below five years of age, living in conditions of crowding [5,6], soldiers serving under field conditions, men who have sex with men, and travelers to LMICs [1,7,8].

An emerging global increase in the antimicrobial resistance of *Shigella* narrows the antibiotic treatment options, especially in young children [9,10,11].

The primary prevention of shigellosis is based on universal access to safe water and improved sanitation, in combination with personal and food hygiene. The low inoculum required to cause shigellosis (100–1000 organisms) facilitates transmission of the disease and explains the frequent failure of preventive sanitary and hygiene measures even in high-income countries [5,10,12]. An efficacious vaccine would therefore complement and accelerate disease burden reduction, particularly in LMICs and other highly endemic locations where primary preventive methods are practically unattainable in the short to medium term.

The genus *Shigella* includes four serogroups, which are classified based on the structure of the O-SP: including *S. dysenteriae* (group A, 15 serotypes), *S. flexneri* (group B, 17 serotypes and subserotypes), *S. boydii* (group C, 19 serotypes), and *S. sonnei* (group D, 1 serotype). *S. flexneri* and *S. sonnei* serogroups account for nearly 90% of cases of shigellosis worldwide [10,13]. In LMICs, *S. flexneri* is predominant [11,13] while in high-income countries, *S. sonnei* causes the great majority of cases of shigellosis [5,10]. *S. flexneri* serotypes, other than *S. flexneri* 6, share a common backbone structure that consists of tetrasaccharide repeats of three rhamnose residues linked to one N-acetylglucosamine. *S. flexneri* 6 has D-galactose as the third sugar of the tetrasaccharide and N-acetylgalactosamine as the terminal residue. Cross-reactivity between *S. flexneri* 2a and 3a and other *S. flexneri* (other than serotype 6) was demonstrated in guinea pigs [14,15]. The O-antigen repeat of *S. sonnei* single serotype is a disaccharide (FucNAc, 2-acetamido-4-amino-2,4,6-trideoxy-D-galactose; AltUA, 2-amino-2-deoxy-L-altruronic acid).

*Shigella* infection confers serotype-specific immunity as revealed by observational studies and challenge models in primates and humans, thus indicating that the O-SP is the protective antigen. The homologous protection of approximately 70% is short lasting and is probably achieved after repeated contacts with *Shigella* O-SP antigens [5,16,17,18,19]. Taking natural infection as a reference, it is expected that an acceptable *Shigella* candidate vaccine would induce an immune response oriented to O-SP, comparable or stronger than that conferred by natural infection. In line with the worldwide distribution of *Shigella* serotypes and supposing that the cross-protection among *S. flexneri* serotypes in guinea pigs [15] is similar in humans, it has been recommended that a tetravalent vaccine incorporating *S. sonnei* and *S. flexneri* 2a, 3a, and 6 O-antigens could provide an overall coverage for up to 85–90% of the most common *Shigella* serotypes [10,14].

## 2. *Shigella* Vaccine Development and the Emergence of Injectable *Shigella* Conjugate Vaccines into the Scene

*Shigella* vaccine development strategies of the last 60 years included two major approaches to deliver the protective *Shigella* LPS antigen to the host immunocompetent cells: live-attenuated vaccine strains and inactivated *Shigella* vaccine candidates (whole cell or subunit).

### 2.1. Live-Attenuated Shigella Vaccines

Orally delivered, live-attenuated *Shigella* vaccines have been developed with the intention to mimic natural *Shigella* infection and induce a protective local immune response against shigellosis, a disease of the gut mucosa. Advances in recombinant DNA technology and more recently whole genome sequencing of shigellae enabled the development of live-attenuated oral *Shigella* candidates with defined deletions of virulence factors. These replaced the older attenuated mutants obtained by serial passages in vitro, which showed a certain degree of protection in field studies but required the administration of annual boosters and were associated with a risk of reversion to the virulent phenotype [20,21]. The knowledge on the exact changes in the bacterial genome associated with different levels of attenuation together with the various advantages of the manufacturing and delivery of oral live attenuated vaccines strongly supported the investment of research efforts and funding in the development of this vaccination strategy. This has been the leading approach in *Shigella* vaccine development at the Walter Reed Army Institute of Research (WRAIR), Center for Vaccine Development (CVD) at University of Maryland, Institut Pasteur, and Karolinska Institute [14,20].

Two major obstacles appeared and significantly slowed down the process of development of these promising candidates. The first was the narrow window between the immunogenicity and safety of these candidates. The development process of the hybrid *E. coli* K12–*S. flexneri* 2a (EcSf2a1 and 2), *S. flexneri* 2a SC602 vaccine strain, *S. dysenteriae* type 1 (SC599 and WRSd1), the series of *S. sonnei* WRSS1, WRSS2, and WRSS3, and the series of CVD 1204, CVD 1207, CVD 1208, and CVD 1208S exemplified this delicate balance along the developmental pathway [20].

A second emerging concern was the discrepancy in the potential protective capacity of these live, rationally attenuated oral vaccine candidates when tested in western volunteers and in individuals in endemic areas [22]. For example, the live-attenuated *S. flexneri* 2a SC602 strain, which was well tolerated and performed excellently in North American volunteers at a single dose of 10^4^ CFUs [23] in terms of replication, immunogenicity, and protection, demonstrated very poor excretion and low immunogenicity in Bangladeshi adults and children [23,24]. The immunogenicity data related to the *S. flexneri* 2a SC602 vaccine strain in Bangladesh corroborated findings on the weaker immunogenicity and efficacy in children in LMICs compared with high income countries experienced with other oral enteric vaccines, including the two licensed live-attenuated rotavirus vaccines [22]. Different hypotheses have been raised to explain the ‘intestinal barrier’ of volunteers in LMICs against this *S. flexneri* 2a SC602 live vaccine strain and other oral enteric vaccines and solutions proposed to overcome this obstacle [22,24]. An encouraging example in this direction is the improvement of the immunogenicity of the live-attenuated *S. sonnei* WRSS1 vaccine strain, which lacks the ability to spread from cell to cell due to the loss of *Vir*G (or *Ics*A). This was achieved in a recent phase I trial in Bangladeshi adults and children aged 5–9 years after raising the number of oral vaccine doses to three and reducing the volume of bicarbonate buffer to neutralize gastric acidity without increasing reactogenicity [25]. The immune responses elicited by WRSS1 were greater than previously seen with SC602 [24].

### 2.2. Serum IgG antibodies to Shigella LPS Are Linked to Decreased Incidence of Homologous Infection

ELISA was employed in conjunction with passive hemagglutination to prospectively detect anti-LPS antibody rise in individuals involved in epidemics of shigellosis that occurred in Israeli military camps [26,27]. Both tests were sensitive and specific in identifying antibody responses to the corresponding *Shigella* LPS. The dynamics of the various immunoglobulin classes after the onset of disease exhibited peak levels of IgA at two weeks post symptoms onset, and a decrease to baseline levels within 10 weeks, while serum IgG levels were highest at 3–4 weeks and decreased thereafter. The levels of IgG at late convalescence were half of the levels detected at early convalescence. The IgM titers displayed a dynamic comparable to that of IgA, but the magnitude was lower [27].

The utilization of ELISA or passive hemagglutination assay after the treatment of sera with 2-mercaptoethanol allowed to measure the serum IgG anti-LPS fraction separately by ELISA or the non-IgM fraction by passive hemagglutination, circumventing overexpression of the pentavalent IgM fraction in the passive hemagglutination test. This approach uncovered the importance of these immunoglobulin fractions, and subsequently their association with protection against shigellosis previously masked by the overexpression of IgM in the old agglutination and hemagglutination assays. The pattern of the IgG subclass rise induced by natural *Shigella* infection was serogroup-dependent. IgG2 was the leading subclass elicited in response to *S. flexneri* 2a infection while IgG1 and IgG2 were the major fractions induced by *S. sonnei* infection [28].

Further seroepidemiological studies undertaken among Israeli soldiers highly exposed to *Shigella* revealed that pre-existent serum IgG antibodies to *S. sonnei* or to *S. flexneri* 2a LPS were strongly associated with a reduced risk of homologous *Shigella* infection [26,29,30]. These studies were carried out in the late 1980s and beginning of the 1990s, when shigellosis was widespread among Israeli recruits serving under field conditions in which *Shigella* species with a very low infectious dose were easily transmitted feco-orally by multiple routes [7,31]. Under these conditions, soldiers with “low” IgG titers to *S. sonnei* LPS at baseline had a 5.5-fold (*p* = 0.0001) higher risk to acquire *S. sonnei* and develop shigellosis versus soldiers with “high” antibody titers. In agreement with these findings, analysis in *S. flexneri* 2a outbreaks showed an odds ratio of 4.3 for ELISA serum IgG titers to *S. flexneri* 2a LPS. These figures correspond to an 82% and 77% reduction in the risk of shigellosis attributed to the homologous serotypes in individuals with high levels of serum IgG antibodies against *S. sonnei* at baseline.

**Figure 1:**
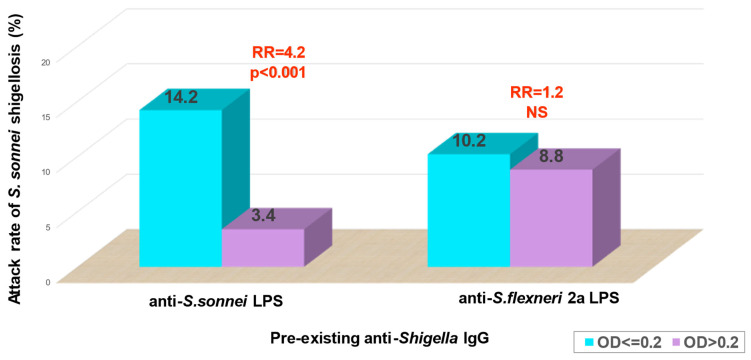
Pre-existing anti-*S. sonnei* LPS antibodies & *S. sonnei* shigellosis; RR= relative risk; NS = not significant; OD = Optical density; Blue bars stand for subjects with anti-*S. sonnei* or anti-*S. flexneri* LPS antibodies equal or lower than OD 0.2; Blue bars stand for subjects with anti-*S. sonnei* or anti-*S. flexneri* LPS antibodies equal or lower than OD 0.2; Purple bars stand for subjects with anti-*S. sonnei* or anti-*S. flexneri* LPS antibodies higher than OD 0.2. Cohen D et al. J. Clin. Microbiol. 1990 [30]; J. Infect. Dis. 1992 [32].

There was no association between “high” baseline antibody titers to *S. sonnei* LPS specific antigen and the risk of *S. flexneri* 2a shigellosis and vice versa [29]. This observation supported the assumption that the protection provided by pre-existing IgG anti-LPS is serotype-specific (Figure 1).

The incidence of *S. sonnei* and *S. flexneri* 2a shigellosis, determined based on visits to the clinic because of diarrhea and a stool culture growing these serotypes, was higher among soldiers with shorter versus longer previous exposure to field conditions (3.3% and 0.05%, respectively, *p* = 0.05). The percentage of persons with antibodies to *S. sonnei* or *S. flexneri* 2a LPS was significantly lower among those who served 0–6 months in the field military units compared to those who served 7–15 months in similar conditions (57.0% and 72.3%, respectively, *p* = 0.001) [32]. Recurring exposures to *Shigella* and natural boosters seemed to speedily elicit high levels of serum IgG anti-*Shigella* LPS and further acquired immunity to shigellosis [26,32].

Analogically, the acquisition of IgG anti-LPS antibodies in an age-related mode might be the result of recurring encounters with *Shigella* LPS and with cross-reacting antigens, even though over a longer time. Similar to capsulated pathogens, such as *Haemophilus influenzae* type b, *Streptococcus pneumoniae*, and *Neisseria meningitidis*, the level of “natural” IgG anti-*Shigella* LPS is inversely associated with the age-specific incidence rates of shigellosis. The disease incidence is low among infants less than six months of age, is highest at 1–4 years of age, and decreases thereafter [33,34]. Elevated levels of serum IgG antibodies to *Shigella* LPS were detected in groups of lower socioeconomic status with an increased risk of encounters with *Shigella* or cross-reacting enteric bacteria earlier in life [30,35]. Moreover, anti-*Shigella* LPS antibodies increased in an age-dependent pattern among populations living in highly endemic regions and this was linked to a reduced likelihood for shigellosis [36,37].

### 2.3. Construction of Detoxified O-SP–Protein Carrier Shigella Conjugates

The findings of the observational studies in Israel pointing out the role of serum IgG anti-*Shigella* LPS antibodies in resistance against disease caused by homologous *Shigella* serotypes, and the development of the groundbreaking effective *H. influenzae* type b conjugate vaccine, provided the solid basis for Robbins and Schneerson to create injectable glycoconjugates containing O-SP of *S. flexneri* 2a, *S. sonnei*, and *S. dysenteriae* type 1 (Shiga) bound to protein carriers [38,39]. Robbins and Schneerson expected that the accomplishments with capsular polysaccharides could be applied to LPS to construct glycoconjugates consisting of *Shigella* O-SP, thus obtaining the T-helper cells support to stimulate a strong anti-O-SP T-dependent immune response [38,39].

This approach was novel for a vaccine against shigellosis, a disease of the intestinal mucosa, and was launched while, as described above, a series of live, rationally attenuated oral vaccine strains were extensively investigated.

Robbins and Schneerson postulated that a critical amount of IgG anti-LPS elicited in serum by an injectable conjugate vaccine exudes onto the epithelium of the gut, and in concert with complement, could result in the killing of shigellae reaching the mucosa [34,39].

The endotoxicity of LPS prevents its use as a parenterally administered immunogen. This obstacle was circumvented by the detoxification of LPS to O-SP and binding to a protein carrier. LPS was extracted by the Westphal and Jann hot phenol method [40] and detoxified O-SP was purified by acid hydrolysis [38,41].

The first *Shigella* conjugate was of O-SP of *S. dysenteriae* type 1 (Shiga) covalently bound to tetanus toxoid (TT) protein carrier. Conjugates were prepared by three methods with adipic acid dihydrazide (ADH) as a linker and their immunogenicity was further assessed in mice. The first two methods used ADH bound to the carbonyls of ketodeoxyoctonate (KDO) at the reducing end of the core [38,42]. The third method used the random cyanogen bromide (CNBr)-mediated derivatization of O-SP with ADH and consequent conjugation to carboxyl groups of TT in which O-SP and TT are linked at multiple sites [38,42]. The antibody response in serum samples of mice injected three times, two weeks apart, was the highest after immunization with the conjugates prepared with multipoint attachments using ADH as a spacer (“lattice”-type conjugates) following each injection [38,42]. Subsequent assessment of adjuvanted formulations of the latter revealed that the use of both alum and monophosphoryl lipid A (MPL) in an oil/water emulsion enhanced the induced anti-LPS antibody response [38,42]. The conjugation approach used with *S. dysenteriae* type I was further employed for the construction and evaluation of *S. flexneri* 2a and *S. sonnei* conjugates [41].

In an effort to reach the highest immunogenicity of the *S. flexneri* 2a LPS-based conjugates, random CNBr-mediated derivatization of DeLPS (LPS detoxified with hydrazine) and O-SP (LPS detoxified with acetic acid) with ADH as linker were conjugated to carboxyl groups of TT and compared [43]. The DeLPS material was of greater molecular weight versus the analogous O-SP but the saccharide:protein molar ratio was higher (3:1 to 5:1 versus 2:1) for conjugates including a O-SP component. Both conjugate formulations induced anti-LPS serum IgG antibodies in mice, but the *S. flexneri* 2a O-SP was a superior T-cell-dependent immunogen through its covalent coupling to TT [43]. The LPS acid-mediated detoxification process was adopted in subsequent developments.

Carriers other than tetanus toxoid were further investigated. *S. flexneri* 2a O-SP conjugates were constructed by means of random conjugation chemistry, utilizing a non-toxic recombinant mutant of the *Pseudomonas aeruginosa* exoprotein A (rEPA). rEPA was selected since the presence of antibodies to this carrier is much less expected in humans in comparison to antibodies to TT or diphtheria toxoid induced by routine national vaccination programs. This carrier can also have medical importance, inducing protective antibodies against opportunistic invasive infections caused by *Pseudomonas aeruginosa* [44]. These conjugates induced anti-LPS IgG antibodies in mice [41]. Moreover, whereas the alum adjuvanted and non-adjuvanted conjugates induced similar anti-LPS IgM antibody levels, the alum adsorbed conjugate elicited a higher level of anti-*S. flexneri* 2a LPS IgG in mice after a third injection [41].

Similarly, the *S. sonnei* O-SP-protein conjugates used random chemical conjugation, ADH as a spacer, and rEPA as the protein carrier. However, whereas hydroxyl groups were involved as ADH anchoring sites in the case of *S. dysenteriae* type 1 and *S. flexneri* 2a O-SP, ADH was directly linked to the carboxyl groups of the *S. sonnei* O-SP LPS. Immunogenicity analysis revealed a trend similar to that of the *S*. *flexneri* 2a conjugates [41]. The conjugation process was modified in an attempt at increasing conjugate solubility, limiting aggregation, and avoiding extensive intramolecular and intermolecular cross-linking. It was found that succinylation of the amino group of the protein carrier increased the conjugation yield and improved the immunogenicity of the conjugates in young, outbred mice [42,45].

### 2.4. Assessment of the Safety, Immunogenicity and Protective Efficacy of the Shigella Conjugate Vaccines in Adults

In a phase I clinical trial conducted in the US, adult volunteers were administered two doses of one of three conjugates, *S. dysenteriae* type 1 O-SP-TT, *S. flexneri* 2a O-SP-rEPA and *S. sonnei* O-SP-rEPA at 25μg of O-SP and 75μg protein carrier [41]. The conjugates (0.5 mL) were injected intramuscularly (IM), 6 weeks apart, with or without alum as adjuvant. They demonstrated a good safety profile and were immunogenic. *S. flexneri* 2a-rEPA and *S. sonnei*-rEPA conjugates induced serum IgG and IgM responses similar or higher than those of young Israeli adults recovering from shigellosis. Unlike the mouse data, there was no enhancement of the IgG and IgM response after the second injection, and no increased antibody response was observed in recipients of the conjugates given with alum [41].

**Figure 2:**
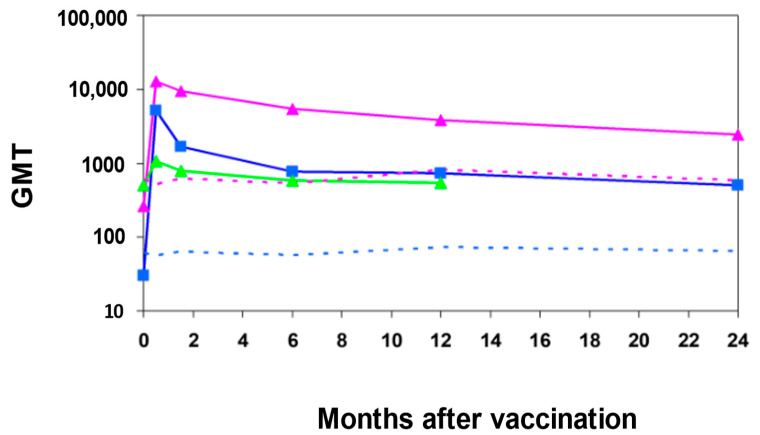
Geometric mean titer of *S. sonnei* LPS antibodies after immunization with one dose of the *S. sonnei*-rEPA conjugate. Pink, blue and green lines stand for IgG, IgA and IgM anti-*S. sonnei* LPS antibodies, respectively. Dotted lines are for hepatitis B vaccinees who served as controls; blue for IgA and pink for IgG anti-*S. sonnei* LPS; GMT = Geometric mean titer. Cohen D. et al. Infect. Immun. 1996 [46].

In a phase II vaccine-controlled clinical trial that assessed the safety and immunogenicity in young adults in Israel, 66 volunteers were vaccinated with *S. sonnei*-rEPA vaccine and 64 received the *S. flexneri*-rEPA vaccine. Of those, 17 and 16 participants, respectively, received a second injection of the same conjugate six weeks following vaccination with the first dose. Sixty two volunteers received a hepatitis B vaccine as control vaccine [46]. Fourteen days post vaccination with the first dose, 90% of *S. sonnei*-rEPA vaccinees and 73–77% of *S. flexneri*-rEPA vaccinees had a four-fold or greater rise in serum IgG and IgA anti-LPS levels, while none of the hepatitis B vaccine recipients presented a significant antibody increase to either LPS. The second dose given on day 42 did not boost antibody titers [46], as was also reported in the phase I trial in the US. Four years after vaccination, 50% of recipients of *Shigella* vaccines still showed four-fold or higher titers, relative to baseline IgG titer. Serum IgG antibody level was the highest and most persistent class of LPS antibodies (Figure 2).

IgA and IgG antibody-secreting cell responses were assessed in a subsample of volunteers. Eighteen out of 23 (78%) volunteers who received *S. sonnei*-rEPA and 13 of 19 (68%) who received *S.* *flexneri*-rEPA had significant IgA-secreting cell responses. Significant IgG antibody-secreting cell responses were detected in 19 of 23 (83%) and 11 of 19 (58%) volunteers following vaccination with *S. sonnei*-rEPA and *S. flexneri* 2a-rEPA, respectively [46].

The increase in serum IgG subclasses in sera of volunteers who were vaccinated with the *S. flexneri* 2a-*r*EPA or *S. sonnei* -*r*EPA vaccine followed a similar pattern to that induced by the corresponding *Shigella* infection. IgG2 was the major subclass induced in response to *S. flexneri* 2a O-SP, whereas IgG1 and IgG2 were the main IgG subclasses elicited in the response to *S. sonnei* LPS O-SP [28,47]. rEPA was the carrier linked to both the O-SPs of *S. sonnei* and *S. flexneri* 2a. Therefore, we assume that the different pattern of IgG anti-LPS subclass response is associated with the chemical structure of the two O-SPs [47].

Interestingly, a significant anti-LPS IgA and IgG response in urine was also observed in 60% of the recipients of the *S. sonnei*-rEPA conjugate [48]. The elicited IgA in urine was of secretory origin and there was a high correlation between the urinary and serum antibody responses [48]. These data suggested that, in addition to a strong serum IgG response, *S. sonnei*-rEPA also induces general mucosal stimulation expressed in the rise of urinary secretory IgA.

**Figure 3:**
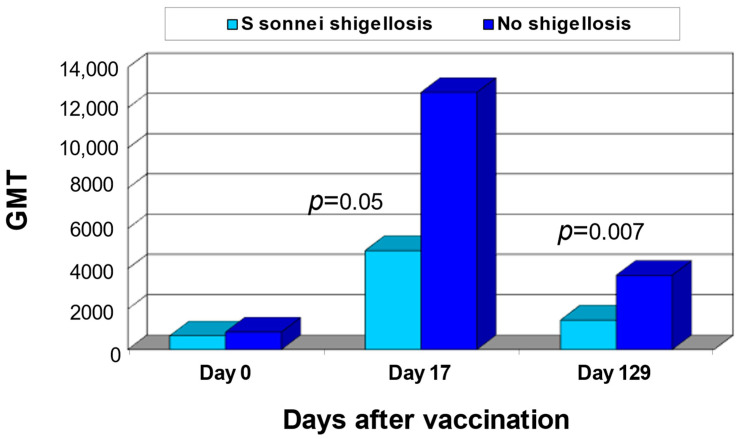
GMTof IgG antibodies to *S. sonnei* LPS among recipients of S *sonnei*-rEPA conjugate in a unit in which an outbreak of *S. sonnei* shigellosis occurred 1–17 days after vaccination. Light blue bars stand for vaccinees who developed *S. sonnei* shigellosis (*n* = 14); blue bars stand for vaccinees who did not develop *S. sonnei* shigellosis (*n* = 109). GMT = Geometric mean titer. Cohen D. et al. Lancet 1997 [49].

A double-blind randomized vaccine-controlled phase III clinical trial evaluated the protective efficacy of a single dose of the *S. sonnei*-rEPA vaccine among 1446 recruits in seven different cohorts (field sites) in Israel at high risk of exposure to *Shigella* [49]. Volunteers were randomly allocated to three vaccine groups and received one injection of *S sonnei*-rEPA and four doses of oral placebo; four oral doses of the hybrid *Escherichia coli* K12–S. *flexneri* 2a (EcSf2a-2) vaccine and one injection of saline (placebo); or one injection of meningococcal control vaccine and four doses of oral placebo. During the follow-up after vaccination, culture-proven cases of *S. sonnei* shigellosis occurred in three cohorts, 70–155 days post-vaccination and in one cohort, as early as 1–17 days post-vaccination. In the first three cohorts, the incidence of shigellosis was 2.2% among vaccinees who received the *S sonnei*-rEPA vaccine versus 8.6% in the control vaccines groups (*p* = 0.006), yielding a protective efficacy of 74% (95% CI 28–100). Moreover, *S sonnei*-rEPA conferred significant protection against culture-proven *S. sonnei* shigellosis in the cohort in which cases occurred 1–17 days post-vaccination, yielding 43% vaccine efficacy (95% CI 4–82), *p* = 0.039 [49]. There were no cases of *S. flexneri* 2a shigellosis among the volunteers during the active surveillance periods. Thus, the efficacy of the EcSf2a-2 vaccine could not be assessed. Pre-vaccination levels of serum IgG and IgA antibodies to *S. sonnei* LPS were comparable in recipients of *S. sonnei*-rEPA vaccine and the control vaccines. *S. sonnei*-rEPA vaccinees who developed *S. sonnei* shigellosis had significantly lower serum IgG responses to the homologous LPS than vaccinees who did not develop *S. sonnei* shigellosis. The higher serum IgG antibody response elicited by the *S. sonnei*-rEPA conjugate vaccine in the group that did not develop shigellosis (*p* = 0.014) confirmed the previous observations concerning the relationship between IgG serum antibody level and protection against homologous disease [26,49] (Figure 3).

### 2.5. Assessment of the Safety, Immunogenicity and Protective Efficacy of the Shigella Conjugate Vaccines in Children

In view of the favorable results of the immunogenicity and protective efficacy of the *S. sonnei*-rEPA conjugate vaccine among young adults, age-descending phase II randomized controlled trials were performed with *S. sonnei* and *S. flexneri* 2a conjugates in Israeli healthy children 4–7 years old [50] and 1–4 years old [51]. Results of these studies showed a very good safety profile of the conjugate vaccines in children, confirming safety data previously generated in young adults.

In the age group 4–7 years, both *S. sonnei* and *S. flexneri 2a O-SP-rEPA* conjugates (at 25 μg saccharide and 75 μg protein concentrations), non-adjuvanted, induced significant responses in serum IgM, IgA and IgG to homologous LPS. The highest and most sustained response was in IgG with a four-fold rise in 96% and 98% of vaccinees who received one dose of the *S. sonnei* or *S. flexneri* 2a conjugate, respectively [50]. The second injection of the conjugates induced a homologous booster rise in children given the *S. flexneri* 2a-rEPA but not *S. sonnei* –rEPA vaccines. The anti-LPS IgG antibody titers measured at six months after the second dose declined but were still significantly higher than the pre-vaccination levels.

In the phase II study in children aged 1–4 years, the *Shigella* conjugates evaluated were *S. sonnei*-O-SP linked to CRM9 and *S. flexneri* 2a O-SP linked to succinylated rEPA. The *S. flexneri* 2a–O-SP rEPA_succ_ conjugate was more immunogenic compared to the respective conjugates containing rEPA and CRM9 as carrier proteins in studies in mice and subsequently in adult volunteers [52]. In contrast, succinylation did not improve the immunogenicity of the *S. sonnei* O-SP when conjugated to either CRM9 or rEPA [52]. *S. flexneri* 2a–O-SP rEPAsucc conjugate was further used in young children.

The *S. sonnei* -CRM9 and *S. flexneri* 2a-rEPA_succ_ elicited a four-fold or greater rise in titer in 92% and 85% of volunteers, after the second injection, respectively. The *S. sonnei* conjugate induced a booster response whereas the rise in anti-LPS IgG antibody level elicited by the second dose of *S. flexneri* 2a conjugate was not statistically significant. Two years later, the GMT of IgG anti-LPS induced by both conjugate vaccines were still significantly higher compared to pre-vaccination.

Pre-vaccination and post-vaccination levels of IgG anti-LPS differed according to age, being lower in children compared to adults. The geometric mean levels of homologous IgG anti-LPS at four weeks following vaccination with the second dose of the *S. sonnei* conjugate vaccine were 48.0, 8.0, and 2.9 in adults, children aged 4–7 years and 1–4 years, respectively. The corresponding levels for *S. flexneri* 2a IgG anti-LPS were 113.0, 48.0, and 40.1 [50,51,52]. In all these trials, no heterologous anti-*S. sonnei* or *S. flexneri* 2a LPS rises were found, confirming the possibility of evaluating the two conjugates in a single trial and having them play the role of control vaccine, each one for the other. All studies also documented a satisfactory response to the carrier proteins used. There is no clear-cut explanation for the variability seen in booster effect of the second dose of each of the *S. sonnei* and *S. flexneri* 2a conjugates in the clinical studies. The variation observed could have been vaccine construct and vaccinee (host)-related, or the result of an interaction between both factors.

In view of these data, a double-blind, randomized and vaccine-controlled efficacy trial of *S. sonnei*–rEPA and *S. flexneri* 2a O-SP-rEPA_succ_ conjugates (non-adjuvanted) was carried out among 2799 healthy children aged 1–4 years in Israel [53]. Two intramuscular injections of the conjugates were administered to children six weeks apart. Serum samples were obtained from randomly selected 10% of the children and IgG anti-LPS levels were measured by ELISA. The number of cases of *S. flexneri* 2a shigellosis was very small, which prevented an efficacy analysis of *S. flexneri* 2a O-SP-rEPA_succ_ in this study. The pooled efficacy of the *S. sonnei* conjugate in the prevention of culture-documented *S. sonnei* shigellosis was 27.5%. Nonetheless, an age-stratified analysis revealed an age-dependent efficacy for children who were vaccinated with the *S. sonnei* conjugate vaccine: 3.8%, 35.5%, and 71.1% (*p* = 0.043) for children aged 1–2 years, >2–3 years, and >3–4 years, respectively. Likewise, an age-dependent rise was evident in serum antibody levels elicited by the *S. sonnei* vaccine. The levels of IgG antibodies in ELISA units (EU) against-LPS of *S. sonnei* were 1.4, 3.71, and 6.38 in the age groups 1–2, >2–3, and >3–4 years, respectively [53]. The efficacy figures paralleled the age-related immunogenicity of the *S. sonnei* conjugate. These “dose–response trend” associations suggest that a defined level of serum IgG anti-O-SP antibodies induces protection against shigellosis [26]. Namely, the threshold level associated with 71% protection in the age group 3–4 years in this trial can provide a reference value when evaluating the immunogenicity of the *S. sonnei* component in the new generation of tetravalent conjugates in infants and toddlers, and for forecasting their efficacy in the pediatric target population. At the age groups and surrogate baseline values deduced from children receiving the *S. flexneri* 2a-rEPA control vaccine, since no paired blood samples were obtained from children in this study, there was no correlation between the fold increase in antibody level of the three age groups and the efficacy of the *S. sonnei* conjugate. Such correlations may occur in older age groups or among adults, where baseline values would be higher and more consistent.

The main findings on the immunogenicity and efficacy of the various Shigella conjugate constructs are summarized in Table 1.

## 3. Bridging from the Classical Conjugates to the Novel *Shigella* O-SP-Based Injectable Vaccines

### 3.1. Clinical Evaluation of the Detoxified O-SP–rEPA Shigella Conjugates Confirmed That IgG Antibodies to Shigella LPS Are Correlates of Protection

An important contribution of the detoxified O-SP–rEPA *Shigella* conjugates clinical evaluation has been the confirmation that IgG antibodies to *Shigella* LPS are correlates of protection with mechanistic capabilities.

Adapting prior definitions and criteria for correlates of protection [54] to shigellosis, we found that serum IgG antibodies to *Shigella* LPS meet all the criteria and can be defined as a correlate of protection [26]. *Shigella* anti-LPS serum IgG antibodies are induced by *Shigella* infection and they have been linked with resistance to shigellosis after natural exposure. Their titers increase in an age-dependent mode in parallel with a significant decrease in the incidence of shigellosis, and they have demonstrated a dose–response association with the protection level achieved by the *S. sonnei* conjugate evaluated in efficacy studies in young adults and children. These collective data have been reemphasized by findings of a recent controlled human infection model (CHIM) study showing that LPS-specific serum IgG responses in bioconjugate vaccine (Flexyn2a) recipients were associated with protection against *S. flexneri* 2a homologous disease [55].

Serum IgG antibodies to *Shigella* LPS also exhibited mechanistic protective capabilities. Using the serum bactericidal assay (SBA) [56,57] and utilizing a stable *S. sonnei* phase 1 strain as a target for SBA, we identified a significant increase in SBA titers in the sera of *S. sonnei*-rEPA vaccinees (GMT = 1472; 95% CI: 690–3143, *n* = 25) three months following vaccination, in comparison to a GMT of 50 (95% CI: 31–80) before vaccination [26]. The titers correlated with the IgG anti-*S. sonnei* LPS titers.

Employing the thiocyanate elution assay to measure avidity [58], we found that sera bactericidal activity had a superior avidity index versus sera with no bactericidal activity on *S. sonnei* (Avidity index: 2.3 vs. 1.8, *p* = 0.048) [26]. Post- but not pre-vaccination sera of children receiving the *S. sonnei* or *S. flexneri* 2a O-SP conjugates in the frame of the age-descending immunogenicity trials blocked the in vitro invasion of Caco-2 cells and inhibited rises in IL-1β and IL-8 mRNA and extracellular cytokine levels [59].

We are currently working to identify thresholds of IgG anti-*S. sonnei* LPS that predicted specific levels of vaccine efficacy in the phase III study in young adults and in the subsequent efficacy trial in children in Israel. Altogether, the data on IgG anti-*S. sonnei* LPS as a correlate of protection stemming from the evaluation of the detoxified O-SP–rEPA *Shigella* conjugates can support and guide the clinical development of the new generation of conjugates, namely bioconjugates and synthetic carbohydrate-based conjugates, or the subunit generalized modules for membrane antigen (GMMA)-based vaccines [55,60,61,62]. It would be of much value to also corroborate these data on serum IgG anti-*S. sonnei* LPS correlate of protection and proposed thresholds in observational studies and clinical trials of the vaccine candidates in LMIC children who are the target for future immunization. We cannot rule out the possibility that a host who has previously experienced mucosal exposure to *Shigella* antigens (e.g., children above three years of age) may be better protected by the parenteral administration of a conjugate vaccine than infants and very young children. Recent studies that we carried out on consecutive plasma samples collected from Zambian infants at the age of 6, 14 and 52 weeks are consistent with early exposure to *Shigella* and indicate naturally acquired IgG and IgA antibodies to *S. flexneri* 2a and *S. sonnei* LPS in a proportion of infants between 14 and 52 weeks of age [63]. At the age of nine months, targeted for the first dose of a *Shigella* vaccine in such settings, the oral natural priming documented by the early rise in anti-LPS antibodies may play a supportive role in the immune response and subsequent protection following the parenteral delivery of immunogenic vaccine candidates.

### 3.2. O-SP Acetylation and Potential Cross-Reactivity/Protection between S. flexneri Serotypes

**Figure 4:**
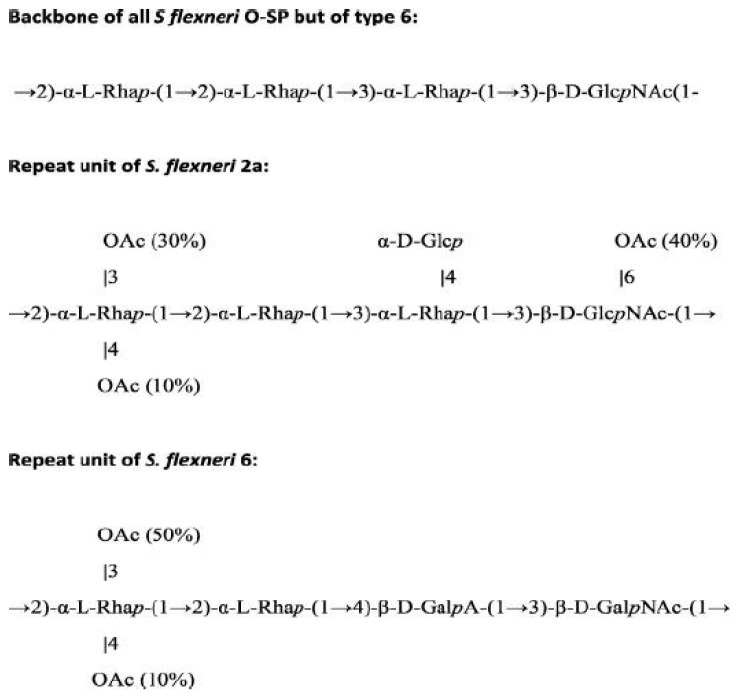
O-SP repeat unit structures of *S. flexneri* 2a and *S. flexneri* 6. Farzam N. et al. Vaccine 2017 [64].

Though there were not enough cases of *S. flexneri* 2a shigellosis to the assess efficacy of the *S. flexneri* 2a-rEPA conjugate against homologous *S. flexneri* 2a shigellosis in the efficacy trial conducted among children aged 1–4-years, the *S. flexneri* 2a conjugate showed a trend of protection (~50%, albeit not reaching statistical significance) against *S. flexneri* serotype 6 infection in the age group 3–4 years [53]. It was hypothesized that the O-acetylated disaccharide rhamnose present on the same position in each one of the two O-SPs backbones, which would otherwise have a different structure, could account for the observed cross-reactivity between *S. flexneri* 2a and *S. flexneri* 6 LPSs. (Figure 4). A more recent study utilizing residual serum samples from *S. flexneri* 2a conjugate vaccinees involved in previous clinical trials demonstrated that 20–32% of adults and children 3–7 years of age receiving one dose or two doses of conjugate, respectively, had a ≥4-fold rise in anti-*S. flexneri* 6 LPS and showed no rise in antibodies to *S. sonnei* LPS, which served as negative control [64]. These and additional data from inhibition studies with homologous and heterologous soluble O-SPs in humans were in disagreement to the findings in mice and guinea pigs that reported a complete absence of cross-reactivity between the two *Shigella* LPSs [15,64]. These original findings have been the basis for studies underway at Tel Aviv University on the potential cross-reactivity between *S. flexneri* 2a and *S. flexneri* 6 LPSs by means of binding and functional antibodies, maintaining a two-side hypothesis concerning the role of acetylation of the O-SP in the vaccines and/or in the in the coating LPS. Significant cross-reactivity and subsequent potential cross-protection between these most common *S. flexneri* serotypes could perhaps allow a reduction in the number of costly multivalent vaccine components.

### 3.3. Efforts to Further Enhance the IgG Response to Shigella O-SP-Protein Conjugates

In search for a more immunogenic conjugate that can elicit a critical IgG anti-LPS level to confer protection in infants and young children, it was found that protein conjugates of synthetic saccharides could induce higher levels of serum IgG LPS antibodies in mice than those of the O-SP from *S. dysenteriae* type 1 [65].

The O-SP of *S. dysenteriae* type 1 (approximately 27 tetrasaccharide repeat units), prepared by acid hydrolysis of the LPS, was bound to human serum albumin (HSA) by multiple point attachment (O-SP-HSA): The molar ratio of HSA to O-SP was 1.0. Synthetic saccharides, composed of one or multiples of the O-SP tetrasaccharide, equipped with a spacer at their reducing end, were bound to HSA by a single point attachment: The average molar ratios of the saccharides to HSA ranged from 4 to 24. The conjugates of the octamer, dodecamer, and hexadecamer synthetic saccharides elicited IgG LPS antibodies after the second injection, a statistically significant rise (booster) after the third injection, and higher levels than those vaccinated with O-SP-HSA (*p* = 0.0001). The highest geometric mean levels of IgG anti-LPS were elicited by the hexadecamer with nine chains or 9 moles of saccharide/HSA (15.5 ELISA units), followed by the octamer with 20 chains (11.1 ELISA units) and the dodecamer with 10 chains (9.52 ELISA units) [65].

This breakthrough finding in mice on the immunogenicity of protein conjugates of the *S. dysenteriae* type 1 synthetic saccharides was a landmark for the development of the novel *Shigella* synthetic carbohydrate-based conjugates at Institut Pasteur [42,66]. *S. flexneri* 2a-TT conjugate (Sf2a-TT15) original synthetic oligosaccharide-protein conjugate administered at two concentrations, alum-adjuvanted and non-adjuvanted, was safe and well tolerated and induced high titers of anti-*S. flexneri* 2a LPS IgG antibodies in a first-in-human phase I study conducted in Israel [60]. The immune response induced by the Sf2a-TT15 first carbohydrate-based synthetic conjugate evaluated in humans was stronger than that elicited by the detoxified *S. flexneri* 2a-rEPA evaluated in a similar young adult population [25].

## 4. Conclusions

The classical detoxified glycoconjugates have changed the paradigm of *Shigella* vaccine development, demonstrating that a parenterally delivered potent vaccine can protect against a mucosal intestinal infection. All stages of the preclinical and clinical development of the classical conjugates provided valuable data that have triggered and guided the development of the current new generation of mono and tetravalent glycoconjugates and other injectable O-SP based *Shigella* vaccine candidates. It is assumed that the lessons learned, the contribution of the new methodological advances, and the current financial support will lead to the licensure of the desired vaccine(s) that will fill the gap and confer protection against shigellosis to infants and very young children in highly endemic regions all over the world.

## Figures and Tables

**Table 1:** *Shigella* conjugate constructs and immunogenicity and efficacy in age descending studies.

Article	Stage in Clinical Development	Vaccine Constructs and Study Groups	Main Findings
Taylor D et al., 1993 [41]	Phase I, safety and immunogenicity, US adult volunteers	Open study *S. dysenteriae* type 1 O-SP-TT, *S. flexneri* 2a O-SP-rEPA and *S. sonnei* O-SP-rEPA at 25 μg of O-SP and 75 μg protein carrier; 2 doses 6 weeks apart	*S. flexneri* 2a-rEPA and *S. sonnei*-rEPA induced serum IgG and IgM responses similar or higher than those of young Israeli adults recovering from shigellosis. No booster effect of 2nd dose. No effect of alum
Cohen D et al., 1996 [46]	Phase II, safety & immunogenicity, Israeli adult volunteers	Randomized, vaccine-controlled, and unblinded study. Volunteers vaccinated with *S. sonnei*-rEPA or *S. flexneri*-rEPA conjugates. A sub-sample received a second injection 6 weeks after first dose. hepatitis B vaccine served as control vaccine	90% of *S. sonnei*-rEPA and 73–77% of *S. flexneri*-rEPA vaccinees had a 4-fold or greater rise in serum IgG and IgA anti-LPS; no similar response found in hepatitis B vaccine recipients. No booster effect of 2nd dose. Two years after vaccination, 50% of recipients of *Shigella* vaccines still showed 4-fold or higher titers, relative to baseline IgG titer. Serum IgG antibody level was the highest and most persistent class of LPS antibodies.
Cohen D et al., 1997 [49]	Phase III, efficacy, Israeli adult volunteers	Double-blind randomized vaccine-controlled trial; single dose of the *S. sonnei*-rEPA vaccine versus the oral hybrid *E. coli* K12–*S. flexneri* 2a (EcSf2a-2) and tetravalent meningococcal control vaccines.	Incidence of *S. sonnei* shigellosis: 2.2% among vaccinees who received the *S sonnei*-rEPA vaccine versus 8.6% in the control vaccine groups (*p* = 0.006); protective efficacy: 74% (95% CI 28–100); Also 43% (95% CI 4–82, *p* = 0.039) protection observed in a cohort in which cases occurred 1–17 days post-vaccination. *S. sonnei*-rEPA vaccinees who developed *S sonnei* shigellosis had significantly lower (*p* = 0.014) serum IgG responses to the homologous LPS than vaccinees who did not develop *S. sonnei* shigellosis reinterring the relationship between IgG level and protection against homologous disease
Ashkenazi S et al., 1999 [50]	Phase II, safety and immunogenicity, Israeli children 4–7 years old	Randomized, vaccine-controlled, and unblinded study. Children vaccinated with *S. sonnei*-rEPA or *S. flexneri*-rEPA or receiving hepatitis B vaccine as control vaccine.	*S. sonnei* and *S. flexneri* 2a O-SP-rEPA conjugates non-adjuvanted, induced significant responses in serum IgM, IgA and IgG to homologous LPS; the highest and most sustained response was in IgG with a fourfold rise in 96% and 98% of vaccinees. A second injection of the conjugates induced a homologous booster rise in children given the *S. flexneri* 2a-rEPA but not *S. sonnei* –rEPA vaccines.
Passwell J et al., 2003 [51]	Phase II, safety and immunogenicity Israeli children aged 1–4 years	Randomized study of *S. sonnei*-O-SP linked to CRM9 and *S. flexneri* 2a O-SP linked to succinylated rEPA; 2 doses 6 weeks apart.	The *S. sonnei* -CRM9 and *S. flexneri* 2a-rEPAsucc elicited a fourfold or greater rise in titer in 92% and 85% of volunteers, after the second injection, respectively. The *S. sonnei* conjugate induced a booster response whereas *S. flexneri* 2a conjugate did not. Two years later, the GMT of IgG anti-LPS induced by both conjugate vaccines were still significantly higher compared to pre-vaccination.
Passwell J et al., 2010 [53]	Phase III, efficacy, Israeli children aged 1–4 years	Double-blind, randomized and vaccine-controlled efficacy trial of *S. sonnei* –rEPA and *S. flexneri* 2a O-SP-rEPAsucc conjugates (non-adjuvanted); 2 doses 6 weeks apart; Blood samples obtained from 10% of participants 2 weeks or more after second dose.	Age-dependent efficacy of the *S. sonnei*–rEPA conjugate against *S. sonnei* shigellosis: 3.8%, 35.5% and 71.1% (*p* = 0.043) for children aged 1–2 year, >2–3 years and the >3–4 year, respectively. The pooled efficacy was 27.5%. Levels of IgG antibodies in ELISA units against-LPS of *S. sonnei* were 1.4, 3.71 and 6.38 in the age groups 1–2, >2–3 and >3–4 years, respectively. The efficacy figures paralleled the age-related immunogenicity of the *S. sonnei* conjugate suggesting that a defined level of serum IgG anti-O-SP antibodies induces protection against shigellosis. There were not enough cases of *S. flexneri* 2a shigellosis to assess the efficacy of the *S. flexneri* 2a O-SP-rEPAsucc conjugate.

## Data Availability

Not applicable.
